# Changes in Soil Microbial Activity, Bacterial Community Composition and Function in a Long-Term Continuous Soybean Cropping System After Corn Insertion and Fertilization

**DOI:** 10.3389/fmicb.2021.638326

**Published:** 2021-04-07

**Authors:** Demin Rao, Fangang Meng, Xiaoyan Yan, Minghao Zhang, Xingdong Yao, Kyung Seok Kim, Jing Zhao, Qiang Qiu, Futi Xie, Wei Zhang

**Affiliations:** ^1^Soybean Research Institute, Shenyang Agricultural University, Shenyang, China; ^2^Jilin Academy of Agricultural Sciences, Changchun, China; ^3^Department of Natural Resource Ecology and Management, Iowa State University, Ames, IA, United States

**Keywords:** soybean, corn insertion, soil bacteria, soil respiration, enzyme activity, NPK fertilization, functional structure

## Abstract

Corn-soybean rotation and fertilization are common practices improving soil fertility and crop yield. Their effects on bacterial community have been extensively studied, yet, few comprehensive studies about the microbial activity, bacterial community and functional groups in a long-term continuous soybean cropping system after corn insertion and fertilization. The effects of corn insertions (Sm: no corn insertion, CS: 3 cycles of corn-soybean rotations and CCS: 2 cycles of corn-corn-soybean rotations) with two fertilization regimes (No fertilization and NPK) on bacterial community and microbial activity were investigated in a long-term field experiment. The bacterial communities among treatments were evaluated using high-throughput sequencing then bacterial functions were predicted based on the FaProTax database. Soil respiration and extracellular enzyme activities were used to assess soil microbial activity. Soil bacterial community structure was significantly altered by corn insertions (*p* < 0.01) and fertilization (*p* < 0.01), whereas bacterial functional structure was only affected by corn insertion (*p* < 0.01). The activities of four enzymes (invertase, β-glucosidase, β-xylosidase, and β-D-1,4-cellobiohydrolase) involved in soil C cycling were enhanced by NPK fertilizer, and were also enhanced by corn insertions except for the invertase and β-xylosidase under NPK fertilization. NPK fertilizer significantly improved soil microbial activity except for soil metabolic quotient (qCO_2_) and the microbial quotient under corn insertions. Corn insertions also significantly improved soil microbial activity except for the ratio of soil induced respiration (SIR) to basal respiration (BR) under fertilization and the qCO_2_ was decreased by corn insertions. These activity parameters were highly correlated with the soil functional capability of aromatic compound degradation, which was the main predictors of bacterial functional structure. In general, the combination of soil microbial activity, bacterial community and corresponding functional analysis provided comprehensive insights into compositional and functional adaptations to corn insertions and fertilization.

## Introduction

Soil bacterial communities play a key role in driving the decomposition of organic matter, nutrient cycling, inhibiting soil-borne diseases and promoting plant growth (Bardgett and van der Putten, 2014), which is beneficial to maintaining soil quality, agricultural sustainability and ecosystem multifunctionality. Ecosystem functions possessed by soil bacterial communities often employed functional metrics like respiration, decomposition, microbial activity or and extracellular enzyme activities (Castle et al., 2017). The composition of soil bacterial community and functional metrics are greatly affected by the corresponding changing edaphic properties, therefore, appropriate agricultural management like irrigation, tillage, crop rotation and fertilizer regime can enable soil microorganisms to perform their diverse ecological functions (Amorim et al., 2020; Kelly et al., 2020).

Soybean continuous cropping is a common practice in Northeast China, whereas soybean yield decreased monotonically with number of years continuously cropped and corresponding deterioration of soil physicochemical properties, accumulation of pathogenic microorganisms, decrease of soil enzymatic activities and the disruption of the soil microbial community (Seifert et al., 2017). Although application of mineral fertilizers, especially nitrogen fertilizers, was the most efficient way of increasing crop yield (Robertson and Vitousek, 2009), the excessive use of chemical fertilizer can easily lead to soil acidification, nitrogen leaching, water eutrophication and lower nutrient use efficiency (Savci, 2012). Crop rotation takes advantage of pre-crop effects as compared to continuous cropping of a single crop, which contributes to improved soil quality and nutrient cycling (Zhao et al., 2009; Gollner et al., 2019). Therefore, corn insertion in a long-term soybean monocropping system with appropriate fertilization may be a solution to decrease the risk of agricultural production (Macholdt et al., 2019).

Zhou et al. (2016) documented that crop type has a great effect on biomass of soil microbes and community structure due to the variations of root exudates and crop residues (Moore-Kucera and Dick, 2008). Crop rotation systems take advantage of the diversity of above-ground crops, and consequently higher efficiency in alleviating obstacles of continuous soybean cropping. Numerous studies found that Crop rotation indirectly regulates the relative abundance of different species in the microbial community through the interaction of soil microorganisms and diverse above-ground plants, which leads to changes in community structure (Navarro-Noya et al., 2013). For instance, the important bacterial phyla such as *Proteobacteria*, *Actinobacteria*, and *Firmicutes* showed a higher relative abundance under wheat-corn-soybean crop rotations than under continuous soybean cropping (Zhu et al., 2014), and the community structure under crop rotation with a higher complexity. In addition, the soil microbial activities (including soil respiration and soil enzyme activities) have been frequently investigated under crop rotation system. A 12-year crop rotation experiment showed that crop rotation increased activities of β-1,4,-glucosidase and β-D-1,4-cellobiohydrolase, and their soil CO_2_ emission experiment showed that rotations increased potential C mineralization by as much as 53% (McDaniel and Grandy, 2016). Therefore, the activities of enzymes and soil respiration determine the intensity of soil biogeochemical processes to some extent. Furthermore, some genes linked to soil functions have also been extensively studied in crop rotation systems. Ammonia-oxidizing bacteria (AOB) and archaea (AOA) that mediated soil nitrification (Ouyang et al., 2018) and the denitrification genes (nirK, nirS, and nosZ) respond differently to rotation regimes (Maul et al., 2019). Additionally, growth of AOA has been linked to NH_3_ derived from organic materials, whereas NH_3_ from ammonium or urea fertilizers typically supports preferential growth of AOB over AOA (Munroe et al., 2016). Although the key soil enzymes or genes related to soil functions had been studied in response to short-term crop rotations (Su et al., 2015), There is still insufficient understanding of the function of the bacterial community at the level of the community, especially the response of the community function structure to the combination of corn insertion and fertilization. Fertilization is an important measures in agricultural production, which will alter the physical structure, nutrient availability and resource heterogeneity of the soil environment (Chen et al., 2020), This in turn will affect the microbial communities that exist in a specific environment, and possibly their roles in ecosystem functioning (Carney and Matson, 2005). Nevertheless, the effect of corn inserted into a long-term soybean monocropping system by different rotation regimes with NPK fertilization on functional composition of soil bacteria remains unclear.

Considering the lack of comprehensive study of how soil properties, microbial activity, bacterial community and functional composition are affected by corn insertion with different rotation regimes and NPK fertilizer in a long-term soybean monocropping system the bacterial community and its functional structure of subsequent soybean were assessed using high throughput sequencing when the continuous cropping soybean field was inserted into the corn crop and treated with NPK fertilization. The activity of soil microorganisms was analyzed by measuring the activity of soil C cycling enzyme and soil respiration. The major objectives were to (i) compare the effects of corn insertion with NPK fertilization on the bacterial community functional structure and microbial activity in a long-term soybean monocropping system; (ii) find the key environmental and microbial activity factors that affect the bacterial community structure and functional structure, respectively; (iii)evaluate the contribution of inorganic fertilizers in shaping the bacterial community and functional structure; (iv) clarify the relationship between soil bacterial community structure, functional structure, soil factors and microbial activity.

## Materials and Methods

### Site Description and Experimental Design

The experiments were conducted at Gongzhuling experimental station of Jilin Academy of Agricultural Sciences, China (43°30′23′′N, 124°48′34′′E, elevation 220 m) in 2012–2017 (Supplementary Figure 1). The field we used had been growing soybeans continuously for 10 years before we started our experiment. The climate in this site belongs to cold temperate continental monsoon climate, with the mean annual precipitation of 450–600 mm, mean annual temperature of 5–6°C and approximately 80% of the total precipitation falls during the growing season from April to September. The soil on this site is classified as light chernozem (Zhao et al., 2017) and originated from the quaternary yellow sediment soil, with approximately 28.7 mg kg^–1^ of available phosphorus (AVP), 169.2 mg kg^–1^ of available potassium (AVK), 175.1 mg kg^–1^ of available nitrogen (AVN), 29.4 g kg^–1^ of soil organic carbon (SOC) and a pH of 6.5.

A split-plot design was used in this experiment and all treatments with three repetitions. Corn insertion treatments were the main plots which included no corn insertion (Sm), 3 cycles of corn-soybean rotations (CS) and 2 cycles of corn-corn-soybean rotations (CCS). Fertilization treatments were the sub-plots included NPK fertilizer and no fertilization (Supplementary Table 1). The chemical fertilizer were applied after the annual corn and soybean harvest. The pure N, P, and K fertilization levels were 150, 75, and 75 kg ha^–1^ year^–1^, respectively. The N, P, and K fertilizers used in this experiment were urea, superphosphate and potassium sulfate, respectively. The soybean and maize were the main local cultivated varieties which were Xianyu 335 and Jiyu 86, respectively.

### Soil Sampling

All soils were collected from plots coming out of the soybean phase in order to avoid confounding effects of current crop differences. Bulk soil samples were collected from each soybean planting plot at the grain-filling stage of soybean in August 2017 and each sample consisted of five cores (5 cm diameter × 20 cm height) that were randomly selected points in each plot, then mixed thoroughly to form a composite sample. The soil sample was sieved with a 2 mm sieve to remove impurities such as leaves, roots and rocks, and then divided into three parts. One part stored at −80°C for later DNA extraction, one part stored at 4°C for microbial activity analysis and the other part was used for the soil property analysis.

### Soil Properties

Soil pH was determined using a fresh soil to water ratio of 1:5 by pH-meter (Mettler Toledo FE20, Shanghai, China). Soil water content (WC) was measured by drying at 105°C to constant weight. Soil nitrate (NO_3_^–^-N) and ammonium (NH_4_^+^-N) were measured using the methods detailed in paper (Sebilo et al., 2004). AVK was extracted with 1 M of ammonium acetate then determined using a flame photometer (FP640, INASA, China). AVP was extracted with 0.03 M hydrochloric acid ammonium fluoride and determined according to the Bray method (Bray and Kurtz, 1945). Total nitrogen (TN) determined by using a Vario MAX CNS elemental analyzer (Elementar, Hanau, Germany). SOC was determined by dichromate oxidation and titration with ferrous ammonium sulfate (Walkley and Black, 1934). Dissolved organic carbon (DOC) were extracted by adding 50 ml of 0.5 M K_2_SO_4_ to 10 g fresh soil, shaking for 1 h, and then vacuum filtering through glass fiber filters (Fisher G4, 1.2 μm pore space) and determined by using a total organic carbon analyzer (Multi N/C 3000; Analytik, Jena, Germany).

### Soil Microbial Activity Parameters

Soil basal respiration (BR) and substrate-induced respiration (SIR) were quantified using airtight incubation jars (2,500 ml) modified with GMP343 CO_2_ probes (Vaisala, Helsinki, Finland) which can real-time monitor the CO_2_ concentration in jars. The empty airtight incubation jars serve as a control. Both BR and SIR were measured on 10 g (oven-dry soil) of fresh soils in airtight incubation jars and brought to 50% water-holding capacity, which is conducive to soil respiration (Grandy and Robertson, 2007). The soil for SR and SIR analysis was received 1ml sterile water and 1ml glucose solution (40 mg/L), respectively, then incubated at 27°C in the dark for 10 days. Both SR and SIR were calculated from the net accumulation of CO_2_ over time and were expressed as mg CO_2_-C kg^–1^ h^–1^. The chloroform-fumigation extraction method was utilized for the analysis of the soil microbial biomass carbon (MBC) which expressed as μg g^–1^ of dry soil (Vance et al., 1987). The metabolic quotient (qCO_2_) was determined by the ratio of the SR to MBC (Dilly and Munch, 1998) which expressed as mg CO_2_ g MBC^–1^ h^–1^. Microbial quotient (MBC:SOC) was the ratio of MBC to SOC (Singh et al., 2016). The ratio of SIR to BR was calculated.

The activities of four enzymes (β-D-1,4-cellobiohydrolase, β-xylosidase and β-glucosidase and invertase) involved in soil carbon cycling were assessed within 48 h of sample collection. The activities of enzymes (β-D-cellulosidase, β-xylosidase and β-glucosidase) were measured using 4-methylumbelliferyl-linked compounds as substrate analogs and the product 4-methylumbelliferyl (MUB) of which fluoresces when released. The detailed methods for the determination of the three enzymes activity was conducted according to Bell et al. (2013), but with some modifications: the pH of sodium acetate buffer was 6.0. The incubation temperature and time were 25°C and 3 h, respectively; The fluorescences were measured using a SpectraMax i3 spectrophotometer (Molecular Devices, Sunnyvale, CA, United States) with 365 nm excitation and 450 nm emission filters. The activities were expressed in units of nmol h^–1^ g^–1^ dry soil. Soil invertase activity was determined with the 3,5-dinitrosalicylic acid method using a sucrose solution as a substrate (Gopal et al., 2007).

### DNA Extractions, PCR Amplification and 16S rDNA Sequencing

DNA from different samples was extracted using the E.Z.N.A.^®^ Soil DNA Kit (D5625-02, Omega, Inc., United States) according to manufacturer’s instructions. The reagent, which was designed to uncover DNA from trace amounts of sample has been shown to be effective for the preparation of DNA of most bacteria. Nuclear-free water was used for blank. The total DNA was eluted in 50 μL of elution buffer and stored at −80°C until measurement in the PCR by LC-Bio Technology Co., Ltd., Hang Zhou, Zhejiang Province, China. The V4 region of the prokaryotic (bacterial and archaeal) small-subunit (16S) rRNA gene was amplified with slightly modified versions of primers 515F (5′-GTGYCAGCMGCCGCGGTAA-3′) and 806R (5′-GGACTACHVGGGTWTCTAAT-3′) (Walters et al., 2016). The 5′ ends of the primers were tagged with specific barcodes per sample and sequencing universal primers. PCR amplification was performed in a total volume of 25 μL reaction mixture containing 25 ng of template DNA, 12.5 μL PCR Premix, 2.5 μL of each primer, and PCR-grade water to adjust the volume. The PCR conditions to amplify the prokaryotic 16S fragments consisted of an initial denaturation at 98°C for 30 s; 35cycles of denaturation at 98°C for 10 s, annealing at 54°C/52°C for 30 s, and extension at 72°C for 45 s; and then final extension at 72°C for 10 min. The PCR products were confirmed with 2% agarose gel electrophoresis. Throughout the DNA extraction process, ultrapure water, instead of a sample solution, was used to exclude the possibility of false-positive PCR results as a negative control. The PCR products were purified by AMPure XT beads (Beckman Coulter Genomics, Danvers, MA, United States) and quantified by Qubit (Invitrogen, United States). The amplicon pools were prepared for sequencing and the size and quantity of the amplicon library were assessed on Agilent 2100 Bioanalyzer (Agilent, United States) and with the Library Quantification Kit for Illumina (Kapa Biosciences, Woburn, MA, United States), respectively. PhiX Control library (v3) (Illumina) was combined with the amplicon library (expected at 30%). The libraries were sequenced either on 250PE MiSeq runs and one library was sequenced with both protocols using the standard Illumina sequencing primers, eliminating the need for a third (or fourth) index read.

### Data Analysis

Samples were sequenced on an Illumina MiSeq platform according to the manufacturer’s recommendations, provided by LC-Bio. Paired-end reads was assigned to samples based on their unique barcode and truncated by cutting off the barcode and primer sequence. Paired-end reads were merged using FLASH. Quality filtering on the raw tags were performed under specific filtering conditions to obtain the high-quality clean tags according to the FastQC (V 0.10.1). Chimeric sequences were filtered using Verseach software (v2.3.4). Sequences with ≥97% similarity were assigned to the same operational taxonomic units (OTUs) by Verseach (v2.3.4). Representative sequences were chosen for each OTU, and taxonomic data were then assigned to each representative sequence using the RDP (Ribosomal Database Project) classifier. OTUs abundance information were normalized using a standard of sequence number corresponding to the sample with the least sequences. Alpha diversity is applied in analyzing complexity of species diversity for a sample through 2 indices, including Chao1 and Shannon (Hill et al., 2003; Chao et al., 2005). All these indices in our samples were calculated with QIIME (Version 1.8.0). Beta diversity analysis was used to evaluate differences of samples in species and functions complexity, which were calculated by principal component analysis (Derksen et al., 1993) and were performed by using CANOCO software4.5 (Microcomputer Power Inc., 2002). The bacterial functional analysis was assessed by the FAPROTAX program^1^ after obtaining the identification and abundance information of the OTUs (Louca et al., 2016). The sequence data were submitted to NCBI Sequence Read Archive^2^ with accession number PRJNA658343.

Two-way analyses of variance (ANOVA) was used to test the significant differences within all treatments using SPSS version 22.0 (SPSS Inc., Chicago, IL, United States). The non-parametric multivariate analysis of variance (adonis) method was used to examine the effects on bacterial communities and functional structure of fertilization and corn insertion (Oksanen et al., 2017). Heatmaps were used to visualize bacterial orders and function groups distribution among all samples. Pearson’s correlation coefficients were used to test relationships among soil properties, microbial parameters, soil enzyme activities and soil bacterial functions. Random forest model was used to identify the main predictors of soil bacterial community and functional structure with the rfPermute package (Archer, 2016) of R statistical software. All variables were standardized by *Z* transformation (mean = 0, standard deviation = 1). The redundancy analysis (RDA) were performed by using CANOCO software 4.5 to determine the most significant soil variables that shaped bacterial community and the most significant microbial activity parameters that shaped bacterial functional structure (Ramette and Tiedje, 2007). The differences in relative abundance of bacterial functions and species in phylum between different treatments were measured at the 95% confidence level (Storey et al., 2004). Linear discriminant analysis effect size (LEfSe) (Segata et al., 2011) was used to find the discriminatory biomarkers at multiple taxonomical levels among different corn insertions, then the algorithm (LDA log score threshold >3.5 and *P* < 0.05) was used.

## Results

### Soil Properties and Productivity

NPK Fertilization did not affect the ratio of C:N, but decreased soil pH value. NPK Fertilization induced significant enhancement in soil AVP (104.9%), AVK (9.3%), NH_4_^+^-N, NO_3_^–^-N (31.1%), DOC (29.8%), SOC (22.0%), and TN (28.6%) content except for AVK, NH_4_^+^-N, and WC under corn insertions treatments (CS and CCS), In addition, NPK fertilization showed higher soybean yield (12.9%) and aboveground biomass (16.8%) than no fertilization treatment (Table 1). When compared with the Sm, both CS and CCS increased soybean yield (26.8 and 39.3%, respectively), and aboveground biomass (13.8 and 19.3%, respectively); the CS and CCS resulted in 11.6 and 15.3% reductions of AVP, respectively; the CCS resulted in 6.64% decrease of AVK. The CS and CCS increased the contents of NO_3_^–^-N (22.2 and 24.3%, respectively), DOC (26.3 and 33.9%, respectively) and the SOC (15.4 and 17.3%, respectively) under NPK fertilization treatment. Corn insertions (CS and CCS) showed lower contents of AVP (12.1 and 16.4%), higher levels of NH_4_^+^-N (29.5 and 18.2%), NO_3_^–^-N (14.2 and 24.2%), DOC (29.4 and 36.9%), SOC (12.4 and 13.6%) and WC (19.4 and 16.8%) in contrast to Sm when not fertilized. The NH_4_^+^-N level was significantly affected by the interaction between corn insertion and fertilization treatment, however, the other soil parameters were less affected by the interaction between corn insertions and fertilization treatment (Table 1).

**TABLE 1:** Soil properties and productivity among previous corn and fertilization treatment.

	**Treatment**	**Sm**	**CS**	**CCS**	**Mean**	**Fertilizer**	**Corn insertion**	Fertilizer*corn insertion
NH_4_^+^-N (mg/kg)	Fertilization	20.4(1.83)a	19.4(1.26)ab	19.3(2.34)ab		ns	ns	*
	No fertilization	16.5(1.18)b	21.3(1.74)a	19.5(1.92)ab				
AVP (mg/kg)	Fertilization	40.9	36.2	34.9	37.3(3)A	**	**	ns
	No fertilization	20.1	17.7	16.8	18.2(2)B			
	Mean	30.5(11.6)a	27.0(10.2)b	25.9(9.9)b				
AVK (mg/kg)	Fertilization	142.8	134.5	127.8	135.0(8.5)A	*	ns	ns
	No fertilization	123.6	126.2	120.9	123.6(5.1)B			
	Mean	133.2(12.6)a	130.4(6.7)ab	124.3(4.7)b				
NO_3_^–^-N (mg/kg)	Fertilization	3.6	4.63	4.48	4.24(0.55)A	**	**	ns
	No fertilization	2.87	3.27	3.56	3.23(0.35)B			
	Mean	3.24(0.5)b	3.95(0.76)a	4.02(0.55)a				
DOC (mg/kg)	Fertilization	157.3	194.8	207.1	186.4(23.8)A	**	**	ns
	No fertilization	117.6	152.2	161	143.6(21.6)B			
	Mean	137.4(23.4)b	173.5(25.7)a	184(25.7)a				
SOC (g/kg)	Fertilization	13.5	15.5	15.8	14.9(1.14)A	**	**	ns
	No fertilization	11.3	12.7	12.8	12.2(0.83)B			
	Mean	12.4(1.25)b	14.1(1.59)a	14.3(1.69)a				
TN (g/kg)	Fertilization	1.58	1.65	1.63	1.62(0.11)A	**	ns	ns
	No fertilization	1.25	1.22	1.32	1.26(0.07)B			
	Mean	1.42(0.21)a	1.44(0.26)a	1.48(0.19)a				
C:N	Fertilization	8.56	9.43	9.7	9.23(0.84)A	ns	*	ns
	No fertilization	9.01	10.43	9.74	9.73(0.79)A			
	Mean	8.79(0.79)b	9.93(0.76)a	9.72(0.52)a				
pH	Fertilization	6.13	6.09	6.15	6.12(0.06)B	**	ns	ns
	No fertilization	6.48	6.39	6.42	6.43(0.06)A			
	Mean	6.3(0.2)a	6.24(0.18)a	6.29(0.16)a				
WC (%)	Fertilization	13.6	13.4	13.2	13.4(1.1)A	ns	ns	ns
	No fertilization	11.3	13.5	13.2	12.7(1.1)A			
	Mean	12.5(1.4)a	13.5(0.8)a	13.2(1)a				
Yield (kg/ha)	Fertilization	2509	2993	3264	2922(358)A	**	**	ns
	No fertilization	2006	2733	3025	2588(462)B			
	Mean	2258(294)c	2863(190)b	3144(179)a				
Biomass (kg/ha)	Fertilization	9396	10077	10516	9996(562)A	**	**	ns
	No fertilization	7335	8966	9381	8561(1006)B			
	Mean	8365(1177)b	9522(697)a	9948(704)a				

*AVP, available phosphorus; AVK, available potassium; NH4^+^-N, ammonium nitrogen; NO_3_^–_^N, nitrate nitrogen; DOC, dissolved organic carbon; SOC, soil organic carbon; TN, total nitrogen; WC, water content; no corn insertion (Sm), 2 cycles of corn-corn-soybean rotations (CS), 3 cycles of corn-soybean rotations with fertilizer (CCS); data are means (n = 3), values in parentheses represent standard deviation of the mean (n = 3). Different lowercase letters after values represent significant differences among all treatments based on Duncan’s test (P < 0.05). Different capital letters after values represent significant differences among treatments within NPK fertilization treatments based on Duncan’s test (P < 0.05)*. *P < 0.05; **P < 0.01.

### Soil Bacterial Diversity

The Shannon index was significantly affected by the interaction between corn insertion and fertilization treatment, however, the Chao1 index was less affected by this interaction. When compared with no fertilization, the NPK significantly decreased the values of Chao1 (7.1%) and Shannon diversity, except for the Shannon diversity in CS (Table 2). There were no significant differences in Chao1 index among corn insertions regardless of fertilization, however, the CCS showed a higher value of Shannon than Sm regardless of fertilization and the CS showed a lower value of Shannon than Sm (Table 2).

**TABLE 2:** Soil bacterial richness and diversity across previous corn and fertilization treatment.

	**Treatment**	**Sm**	**CS**	**CCS**	**Mean**	**Fertilizer**	**Corn insertion**	Fertilizer*Corn Insertion
Chao1	Fertilization	3127	3048	3073	3083(68)B	**	ns	ns
	No fertilization	3345	3315	3295	3319(119)A			
	Mean	3236(172)a	3182(178)a	3184(129)a				
Shannon	Fertilization	9.48(0.11)c	9.42(0.03)cd	9.36(0.02)d		**	**	**
	No fertilization	9.71(0.04)b	9.51(0.04)c	9.87(0.07)a				

*Data are means (n = 3), values in parentheses represent standard deviation of the mean (n = 3). Different lowercase letters after values represent significant differences among all treatments based on Duncan’s test (P < 0.05). Different capital letters after values represent significant differences among treatments within NPK fertilization treatments based on Duncan’s test (P < 0.05)*. *P < 0.05; **P < 0.01.

### Soil Bacterial Community and Functional Prediction

**FIGURE 1:**
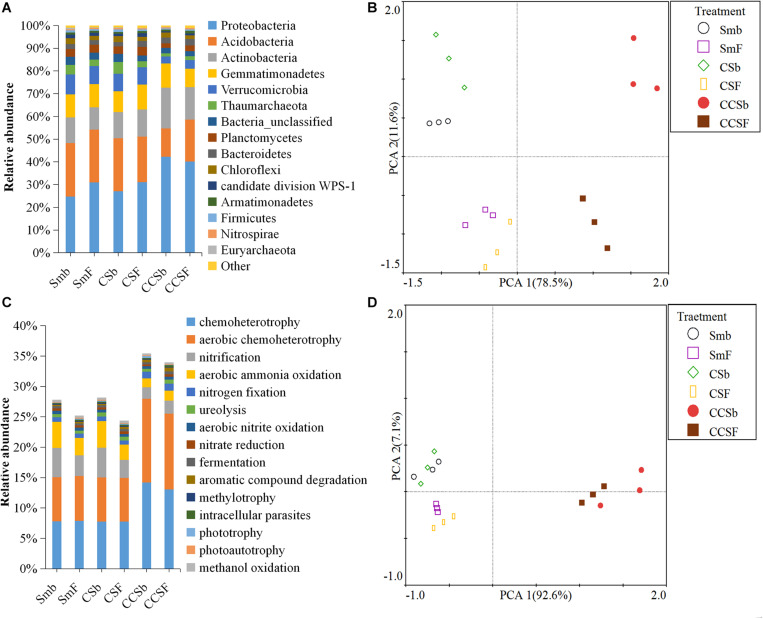
Stacked bar chart of dominant bacteria phyla **(A)** and dominant function group **(B)**. Principal component analyses (PCA) of the bacterial community **(C)** and functional structure **(D)**.

**FIGURE 2:**
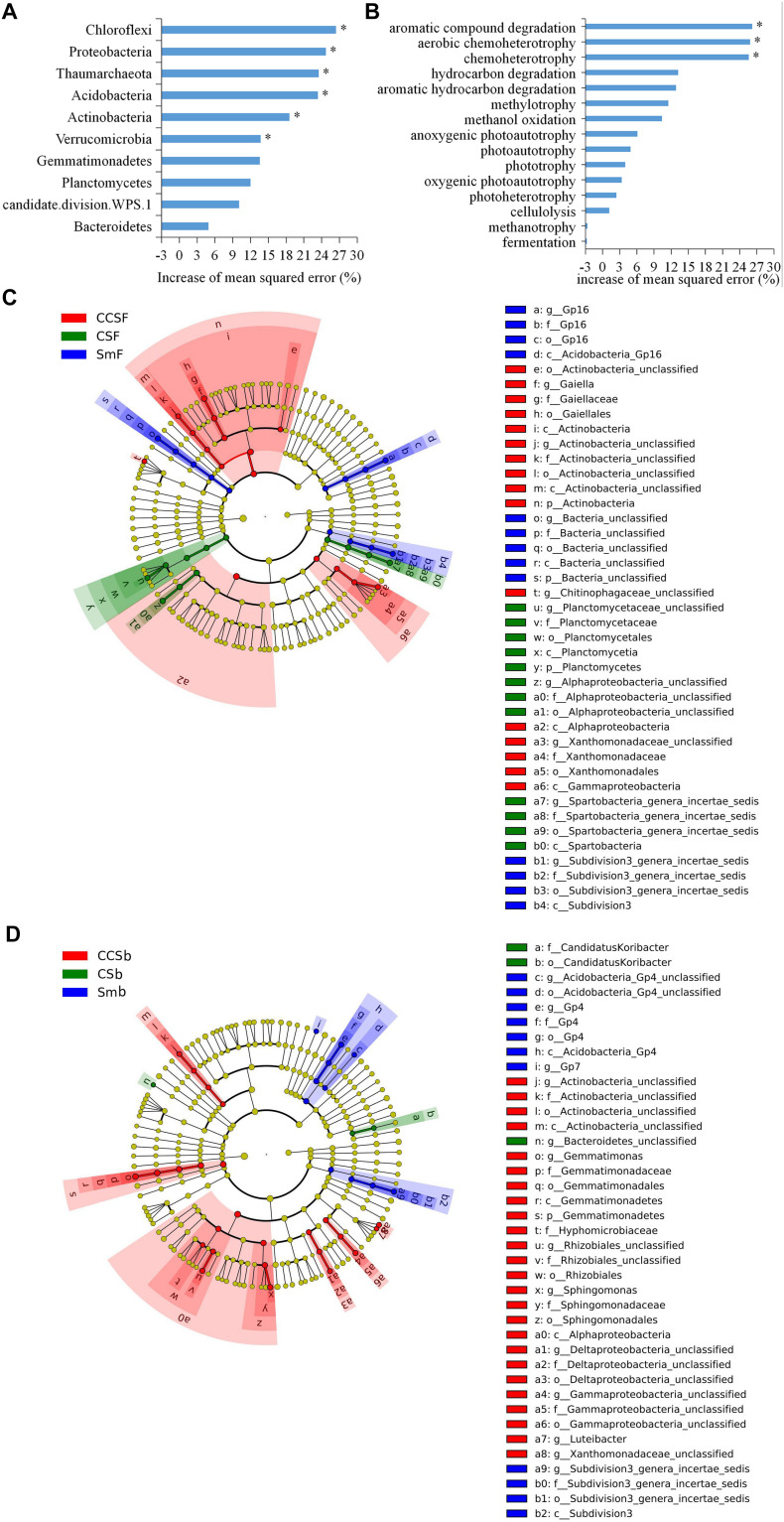
Random Forest mean predictor importance (% percentage of increase of mean square error) of major bacterial phyla studied as predictors of soil bacterial community **(A)** and the major function groups studied as predictors of soil functional structure **(B)**. Significance levels are as follows: *P < 0.05. LEfSe of the bacterial communities under fertilization treatment **(C)** and fertilization treatment **(D)** with an LDA score higher than 3.0. Cladograms indicate the phylogenetic distribution of microbial lineages associated with corn insertions. Circles represent phylogenetic levels from kingdom to genus.

A total of 5,466 OTUs were detected in all soil samples, and most of them were assigned to *Proteobacteria* (23.4 ∼ 42.9%), *Acidobacteria* (11.8 ∼ 24.6%), *Actinobacteria* (9 ∼ 18.6%), and *Gemmatimonadetes* (7.7 ∼ 11.5%) at the phylum level (Figure 1A). Principal component analysis (PCA) clearly showed that the first two components explained 78.5 and 11.6% of the total variability, respectively, and treatments incorporated with NPK fertilizer were separated from treatments with no fertilizer along the second principal component axis. The CCS treatment were further separated from Sm than CS treatment whether fertilized or not (Figure 1B). It was further confirmed by the result that both corn insertions (*R*^2^ = 0.792, *p* = 0.001) and NPK fertilization treatment (*R*^2^ = 0.042, *p* = 0.002) significantly changed the bacterial community (Supplementary Table 2). NPK fertilization showed a higher relative abundance of *Proteobacteria*, but a lower relative abundance of *Chloroflexi* than no fertilizer. Both CS and CCS had a higher relative abundance of *Proteobacteria*, but a lower relative abundance of *Verrucomicrobia* than Sm when not fertilized, however, there was no significant difference in the relative abundance of soil bacteria at phyla level between Sm and CS when fertilized. The CCS had a higher relative abundance of *Proteobacteria* and *Actinobacteria*, but a lower *Acidobacteria* and *Verrucomicrobia* than Sm and CS when fertilized (Supplementary Figures 2A, 3). We performed linear discriminant analysis (LDA) effect size analysis (LEfSe) to identify discriminatory biomarkers (LDA scores of >3) among different corn insertions (Figures 2C,D). The results showed that three bacterial classes including *Actinobacteria*, *Alphaproteobacteria* and *Gammaproteobacteria* had higher relative abundances in CCS system with NPK fertilizer. Three bacterial families including *Xanthomonadaceae*, *Gaiellaceae*, and *Actinobacteria_unclassified* had higher relative abundances in CCS system with NPK fertilizer. Two bacterial classes including *Planctomycetia* and *Spartobacteria* had higher relative abundances in CS system with NPK fertilizer. Two bacterial families including *Spartobacteria_genera_incertae_sedis* and *Planctomycetaceae* had higher relative abundances in CS system with NPK fertilizer. Two bacterial classes including *Subdivision3* and *Acidobacteria_Gp16* had higher relative abundances in Sm system with NPK fertilizer. Two bacterial families including *Subdivision3_genera_incertae_sedis* and *Gp16* had higher relative abundances in Sm system with NPK fertilizer (Figure 2C). When without fertilizer, the discriminant biomarkers enriched in CCS system included members from the classes *Alphaproteobacteria*, *Gemmatimonadetes*, *Actinobacteria_unclassified* and six members from the families *Gammaproteobacteria_unclassified*, *Deltaproteobacteria_unclassified*, *Sphingomonadaceae*, *Rhizobiales_unclassified*, *Hyphomicrobiaceae*, *Gemmatimon- adaceae*. The discriminant biomarkers enriched in CS system included members from the family *CandidatusKoribacter*. The discriminant biomarkers enriched in Sm system included members from the classes *Subdivision3*, *Acidobacteria_Gp4* and two members the families *Gp4* and *Subdivision3_genera_incertae_sedis* (Figure 2D).

Using random forest modeling, we identified the major function group for predicting bacterial functional structure. The results showed that the top 10 phyla of soil bacterial explained 51.13% of the variation of soil bacterial community structure, and the *Chloroflexi, Proteobacteria, Thaumarchaeota, Acidobacteria, Actinobacteria*, and *Verrucomicrobia* as the main predictors of bacterial community (Figure 2A).

FAPROTAX analysis was adopted to predict functions of soil bacterial community. A total of 91 functional categories were linked to the bacterial community in soil and 50 functional groups were obtained by using FAPROTAX. The dominant functional groups were chemoheterotrophy (9.71%), aerobic chemoheterotrophy (9.18%), nitrification (3.37%), and aerobic ammonia oxidation (2.89%) (Figure 1C). Principal component analysis (PCA) clearly showed that the first two components explained 92.6 and 11.6% of the total variability, respectively, and the Sm and CS treatments were grouped well but far away from that of CCS. The treatments with fertilizer were not separated from their correspond treatments without fertilizer (Figure 1D). It was further confirmed by the result that corn insertions treatment (*R*^2^ = 0.637, *p* = 0.003) significantly changed the bacterial functional structure, however, NPK fertilization treatment (*R*^2^ = 0.035, *p* = 0.231) did not significantly affect functional structure (Supplementary Table 2). NPK fertilization treatment had a higher abundance of groups capable of (aerobic) chemoheterotrophy, but a lower abundance of groups capable of nitrification and aerobic nitrite oxidation than no fertilizer under Sm and CS; However, NPK fertilization showed a lower relative abundance of groups capable of (aerobic) chemoheterotrophy and higher relative abundance of groups capable of ureolysis and aromatic hydrocarbon degradation under CCS. The CS enhanced soil N-related functions regardless of fertilization, however, the CCS weakened functions capable of nitrification and aerobic nitrite oxidation, and showed a higher relative abundance of (aerobic) chemoheterotrophy than Sm (Supplementary Figures 2B, 4).

Using random forest modeling, we identified the major function group for predicting bacterial functional structure. The results showed that the top 15 functional groups of soil bacterial explained 43.96% of the variation in soil bacterial functional structure, and the functions capable of aromatic compound degradation and (aerobic) chemoheterotrophy as the main predictors of bacterial functional structure (Figure 2B).

### Soil Microbial Activity and Enzyme Activities

Soil microbial activity indexs include soil MBC, induced respiration (SIR), BR, and the ratio of SIR:BR (Table 3), all of them the values were increased in the soil with fertilization treatment regardless of corn insertions. The values of SIR increased by NPK fertilization in CS and CCS systems (34.0–38.4%) were greater than that in Sm system (26.2%), the values of SIR increased by NPK fertilization in CS and CCS systems (55.9–89.7%) were lower than that in Sm system (111.7%), the ratio of SIR:BR increased by NPK fertilization in CS and CCS systems (13.1–38.5%) were lower than that in Sm system (59.8%). In addition, corn insertions (CS and CCS) significantly increased value of MBC, SIR, BR and ratio of SIR:BR in contrast to Sm regardless of fertilization (except for the ratio of SIR:BR under fertilization treatment). The qCO_2_ was less affected by NPK fertilization treatment, but reduced by corn insertions of CS (9.3%) and CCS (11.4%). The ratio of MBC:SOC was increased by fertilization under CS (9.2%) and CCS (12.1%) treatments, but less affected by fertilization under Sm treatment (Table 3). The CS and CCS resulted in 14.8 and 20.2% increase in the ratio of MBC:SOC, respectively. The CS and CCS resulted in 9.3 and 11.3% decrease in the value of qCO_2_, respectively.

**TABLE 3:** Effects of previous corn and fertilization treatments on soil microbial activity parameters.

**Microbial activity**	**Treatment**	**Sm**	**CS**	**CCS**	**Mean**	**Fertilizer**	**Corn insertion**	Fertilizer*corn insertion
MBC:SOC (%)	Fertilization	1.91	2.22	2.36	2.16(0.21)A	**	**	ns
	No fertilization	1.81	2.04	2.1	1.98(0.15)B			
	Mean	1.86(0.07)c	2.13(0.12)b	2.23(0.15)a				
qCO2 (mg CO_2_ g^–1^ MBC h^–1^)	Fertilization	5.58	4.99	4.81	5.13(0.39)A	ns	**	ns
	No fertilization	5.31	4.88	4.84	5.01(0.31)A			
	Mean	5.45(0.29)a	4.94(0.22)b	4.82(0.1)b				
SIR (mg CO_2_ kg^–1^ h^–1^)	Fertilization	27.7	31.7	33.9	31.1(2.7)A	**	**	ns
	No fertilization	13.1	16.7	21.7	17.2(3.8)A			
	Mean	20.4(8)c	24.2(8.2)b	27.8(6.6)a				
BR (mg CO_2_ kg^–1^ h^–1^)	Fertilization	1.43(0.04)b	1.72(0.06)a	1.79(0.03)a		**	**	*
	No fertilization	1.08(0.05)d	1.26(0.04)c	1.3(0.03)c				
SIR:BR	Fertilization	19.4(0.96)a	18.4(0.34)a	18.9(0.65)a		**	**	**
	No fertilization	12.1(0.54)d	13.3(0.36)c	16.7(0.77)b				
MBC (mg kg^–1^)	Fertilization	256.6(7.15)c	345.5(11.82)b	372.4(9.18)a		**	**	**
	No fertilization	203.4(5.06)d	257.9(7.05)c	269(4)c				
Invertase (mg day^–1^g^–1^)	Fertilization	9.62	10.33	9.78	9.91(0.64)A	**	**	ns
	No fertilization	6.36	8.68	8.13	7.72(1.16)B			
	Mean	7.99(1.82)b	9.51(1.1)a	8.96(1.08)a				
β -glucosidase (nM h^–1^g^–1^)	Fertilization	119.5	132.5	138.2	130.1(11)A	**	**	ns
	No fertilization	84.9	102.6	117.8	101.7(15.6)B			
	Mean	102.2(20.4)c	117.5(17.6)b	128(13.3)a				
β -D-1,4-cellobiohydrolase (nM h^–1^g^–1^)	Fertilization	25.3	28.7	29.4	27.8(2.8)A	**	**	ns
	No fertilization	19.7	25.2	24.9	23.3(3)B			
	Mean	22.5(3.5)b	27.0(2.7)a	27.2(2.9)a				
β -xylosidase (nM h^–1^g^–1^)	Fertilization	48.5(3.79)a	51.4(2.39)a	49.6(1.32)a		**	**	**
	No fertilization	31.4(1.68)d	38.6(3.37)c	43.9(1.72)b				

*MBC, microbial biomass carbon; qCO_2_, metabolic quotient; BR, BR basal respiration; SIR, soil induced respiration. Data are means (n = 3), values in parentheses represent standard deviation of the mean (n = 3). Different lowercase letters after values represent significant differences among all treatments based on Duncan’s test (P < 0.05). Different capital letters after values represent significant differences among treatments within NPK fertilization treatments based on Duncan’s test (P < 0.05)*. *P < 0.05; **P < 0.01.

NPK fertilization enhanced the activities of invertase (IVE), ß-glucosidase (BG), ß-D-1,4-cellobiohydrolase (CHB) and ß-xylosidase (XYL) involved in the soil C cycling. In addition, the activity of IVE increased by NPK fertilization in Sm system (51.3%) was greater than those in CS and CCS systems (19.0–20.3%), the activity of BG increased by NPK fertilization in Sm system (40.8%) was greater than those in CS and CCS systems (17.4–29.2%), the activity of CHB increased by NPK fertilization in Sm system (28.4%) was greater than those in CS and CCS systems (13.9–18.1%), the activity of XYL increased by NPK fertilization in Sm system (54.6%) was greater than those in CS and CCS systems (13.1–33.1%). Both CS and CCS enhanced the four enzymes’ activities except for the IVE and XYL under NPK fertilization. In addition, the significant difference was observed in activity of XYL between CS and CCS (CCS was 13.8% higher than CS) when not fertilized (Table 3).

### Correlation Between Bacterial Community, Soil Properties and Microbial Activity

Pearson correlation analysis was used to assess the correlation of soil variables, microbial activity and enzyme activity with the main predictors of soil bacterial community and functional structure (Table 4 and Supplementary Table 3). Pearson’s correlation analysis showed that the MBC was significantly positively correlated with the function groups capable of C degradation; the microbial quotient (MBC:SOC) was significantly positively correlated with the functional groups except for the group capable of anoxygenic photoautotrophy. The qCO_2_ was significantly negatively correlated with the function groups except for group with function of fermentation and aromatic hydrocarbon degradation. Both BR and SIR were significantly positively correlated with the group capable of fermentation and C degradation; The ratio of SIR:BR was only significantly positively correlated with the function group capable of aromatic compound degradation; The relative abundance of group capable of C degradation were significantly positively correlated with the activity of BG and CHB, and the activity of XYL have a significant correlation with function group with aromatic compound degradation (Table 4).

**TABLE 4:** Pearson’s correlation coefficients for bacterial functional groups, soil microbial parameters, and four carbon metabolism enzyme.

**Function groups**	**MBC**	**MBC:SOC**	**qCO2**	**BR**	**SIR**	**SIR:BR**	**IVE**	**BG**	**XYL**	**CBH**
Chemoheterotrophy	0.38	0.538*	−0.529*	0.244	0.275	0.301	0.031	0.406	0.249	0.273
Aerobic chemoheterotrophy	0.371	0.530*	−0.530*	0.234	0.266	0.294	0.022	0.397	0.241	0.264
Fermentation	0.519*	0.515*	−0.203	0.491*	0.468*	0.355	0.387	0.510*	0.358	0.465
Aromatic compound degradation	0.712**	0.737**	−0.484*	0.631**	0.608**	0.482*	0.4	0.675**	0.483*	0.602**
Hydrocarbon degradation	0.704**	0.734**	−0.569*	0.594**	0.549*	0.413	0.414	0.617**	0.428	0.588*
Anoxygenic photoautotrophy	0.165	0.374	−0.655**	−0.018	−0.151	−0.238	0.019	0.064	−0.039	0.209
Aromatic hydrocarbon degradation	0.693**	0.648**	−0.434	0.625**	0.548*	0.353	0.392	0.578*	0.391	0.567*

IVE, invertase; BG, β-glucosidase; XYL, β-xylosidase; CBH, β-D-1,4-cellobiohydrolase. *P < 0.05, **P < 0.01.

The NO_3_^–^-N, DOC, SOC, and TN contents were significantly negatively correlated with the relative abundances of Thaumarchaeota and Chloroflexi. The dominated phylum Proteobacteria was significantly positively correlated with soil DOC content. The *Actinobacteria* and *Verrucomicrobia* were significantly negatively correlated with the AVK and DOC contents, respectively. The Chao and Shannon index were significantly positively correlated with soil pH, but negatively correlated with AVP, NO_3_^–^-N, DOC, SOC, and TN contents. All the soil microbial activity parameters (except for qCO_2_) were significantly positively correlated with NO_3_^–^-N, DOC, SOC, and TN, but negatively correlated with soil pH. The qCO_2_ was negatively correlated with the ratio of C:N (Supplementary Table 3).

**FIGURE 3:**
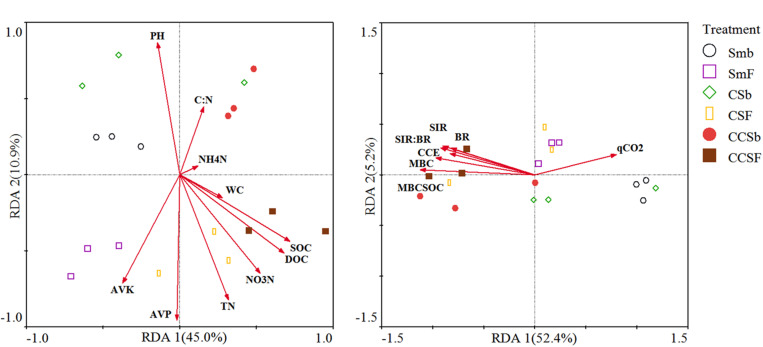
Redundancy analysis (RDA) of soil bacterial community structure associated with soil properties **(A)**, and soil bacterial functional structure associated with microbial activity **(B)**. CCE: the PC1 of principal coordinate analysis of IVE, BG, XYL and CHB which was defined as soil carbon cycling enzyme activities.

We used redundancy analysis (RDA) to assess the effects of soil properties and microbial activity on the compositions of the bacterial community and functional (Figure 3). For the compositions of bacterial community, the first two axes together explained 53.7% of the total variation in bacterial communities. The value of SOC (*F* = 5.54, *P* = 0.017) and AVK (*F* = 4.54, *P* = 0.027) were positively correlated with the bacterial community (Figure 3A). For the compositions of bacterial functional, the first two axes together explained 55.2% of the total variation in bacterial functional compositions. The value of MBC:SOC (*F* = 9, *P* = 0.006) and BR (*F* = 4.46, *P* = 0.036) were positively correlated with the bacterial functional structure (Figure 3B).

## Discussion

In this study, we found that soil quality and nutrient level were increased after inserting corn in long-term continuous soybean cropping system, therefore, corn insertions promoted the increasing of soil productivity of long-term soybean continuous cropping land (Table 1). Unlike fertilization that directly improves soil fertility, corn insertions take advantage of rotation effects as compared to continuous cropping of a single crop, at the same time, soil bacterial community play an important role in balance of soil quality, and the positive effect of corn soybean rotation on soybean root nodule nitrogen fixation was mainly due to the improving soil quality (Ferreira et al., 2000). Dai et al. (2018) showed that NPK fertilization decreased the soil bacterial diversity. Although our fertilization treatment performed the same result, the NPK fertilization had no significant effect on soil bacterial diversity of CS system, perhaps the corn inserted into a long-term soybean monocropping system by CS rotation has a more complex microbial co-occurrence network, which may be reduced the impact of fertilization on soil bacterial diversity (Banerjee et al., 2019). Our results confirmed the result that NPK fertilization significantly decreased soil pH (Ahmad et al., 2014), however, corn insertions had no effect on pH value. In addition, both soil bacterial richness and Shannon index had a significant positive correlation with soil pH (Supplementary Table 3), which indicated that not only pH was an important factor in regulating the composition of bacterial communities (Rousk et al., 2010) but also some other edaphic factors changed by corn insertions may be important to the composition of bacterial communities.

In this study, corn insertions and chemical fertilizer not only increased crop productivity and soil nutrients, but also altered the soil bacterial communities. Consistent with most results that microorganisms in the phyla *Acidobacteria* and *Proteobacteria* usually accounted for most of the soil bacterial community (Fierer, 2017). The *Chloroflexi* showed a highest increase of mean square error according to the ranking of importance in random forest model, then followed by the phyla *Proteobacteria*, *Thaumarchaeota, Acidobacteria*, *Actinobacteria*, and *Verrucomicrobia*, which implied they were the main predictor of the soil bacterial community (Figure 2A). The phyla *Chloroflexi* and *Acidobacteria* belong to oligotrophic groups with slower growth rates that were more likely to decline under nutrient-rich conditions (Ling et al., 2017). This explained the decrease of relative abundance of *Chloroflexi* and *Acidobacteria* under NPK fertilizer and CCS system, in addition, the significant negative correlation between the relative abundance of *Chloroflexi* and soil nutrient indexes confirmed this result, this further indicated the reason of the CCS system with a higher soil productivity (Table 1 and Supplementary Table 3), at the same time, the phyla *Chloroflexi* and *Acidobacteria* can be good indicators of soil condition. The phylum *Proteobacteria* belong to a copiotrophic group with fast growth rates that would likely increase with N addition (Ramirez et al., 2010), so it is related to the availability of soil nutrients, especially the metabolic substrates of microorganisms such as easily decomposable organic compounds. Both corn insertions and fertilization had the effect of increasing soil organic matter (SOC) or DOC, so NPK fertilization increased the content of soil organic matter more significantly in Sm system than that in corn insertions of rotation system (Table 1). This explained the reason that NPK fertilization soil showed a higher relative abundance of *Proteobacteria* under continuous soybean cropping system (Sm), and corn insertion by CCS rotation showed a higher relative abundance of *Proteobacteria* as compared to Sm regardless of fertilization (Supplementary Figure 3), The significant positive correlation between the relative abundance of *Proteobacteria* and soil DOC contents also corroborated this point (Supplementary Table 3). Finally, one of the predominant phyla *Actinobacteria* with ecological function capable of decomposing crop straw and complex polymers (Fan et al., 2014; Bhatti et al., 2017) was more abundant in corn insertion (CCS) in contrast to Sm system regardless of fertilization (Supplementary Figure 3), which may be of key importance in maintaining microbially mediated processes after corn insertion. As revealed by the LEfSe analysis, bacterial communities showed a greater sensitivity to CCS system at all phylogenetic levels regardless of fertilization, however, bacterial communities showed a lower sensitivity to CS system, and moreover the significant differences were only existed at lower phylogenetic levels when not fertilized, which suggested that the distinct compositions of the microflora changed more easily in CCS system than that in CS system. This may be the reason that corn insertion of CCS with more corn continuous insertion into soybean field which weakened the legacy effect of soybean, therefore, the samples of CS were closer to samples of Sm when compared to CCS regardless of fertilization (Figure 1B). Above all, both corn insertions and NPK fertilization significantly affect soil bacterial community (Figure 1B and Supplementary Table 2), and the community-level copiotroph–oligotroph variations corresponding to changing soil nutritional status may be a main driver of soil bacterial community under corn insertion and NPK fertilizer, which is in line with our redundancy analysis result (Figure 3A).

The strength of some soil ecological functions can be reflected to some extent by microbial activity, therefore, soil respiration, qCO_2_, microbial quotient (MBC:SOC) and extracellular enzyme activities were used to make a comprehensive evaluation of microbial activity. As our results reveal that the fertilized soil had a higher microbial activity (Table 3), the abundant organic substrates derived from plants may be a key factor, which means the microbial community formed under NPK fertilized soil had a faster metabolic response to external carbon sources. Lupwayi et al. (2018) showed that both the soil microbial biomass and microbial activity had a significant positive correlation with the SOC content, Our Pearson’s correlation results corroborated this point (Supplementary Table 3). In addition, NPK fertilization only increased the microbial quotient (MBC:SOC) under corn insertions (CS and CCS), but showed no significant effect on qCO_2_ (Table 3). This indicated that the soil microorganisms under corn insertions (CS and CCS) were more efficient in using carbon source substrates for their proliferation in contrast to Sm, in addition, NPK fertilization may had little effect on the proportion of microorganisms with different degree of oxygen demand, which could stabilized the ratio of CO_2_ emitted by microorganisms to microbial biomass. Corn insertions (CS and CCS) significantly increased the value of most microbial activity indicators in contrast to Sm, however, showed no effect on the ratio of SIR:BR under fertilization treatment, and decreased the value of qCO_2_ regardless of fertilization (Table 3). This indicated that microorganisms in CS and CCS systems were more responsive to exogenous carbon addition when not fertilized; corn insertions may had a great effect on the proportion of microorganisms with different degree of oxygen demand, which played a key role in reducing qCO_2_, thus enabling the microbial communities to use more organic substances for growth rather than breathing consumption. This may relate to the lower energy budget of corn insertions (Mäder et al., 2002), and the optimized functional structure of soil bacteria formed through crop rotation that played an important role in reducing soil carbon emissions and maintaining the stability of soil carbon pool (Tiemann et al., 2015). NPK fertilization and corn insertions indirectly can increase soil SOC content by increasing the input of crop residues to soil (Zhang et al., 2018). Crop residues and SOC in farmland significantly affected the biomass and activity of soil microorganisms by changing the substrate supply (Li et al., 2019). Soil extracellular enzymes such as β-D-1,4-cellobiohydrolase (CHB), β-xylosidase (XYL), β-glucosidase (BG), and invertase (IVE) play an important role in the degradation of soil carbon (Liu et al., 2017). Our study showed that NPK fertilization and corn insertions tend to promote the metabolic process of growth of living organisms then promoted the process of soil carbon metabolism (Table 3), which means a higher turnover rates of soil C and this may be important for the accumulation of MBC (Zhang et al., 2019). The significant correlations between these four carbon metabolizing enzymes and the content of soil SOC and DOC confirmed this point (Supplementary Table 3).

The functional diversity of microorganisms enabled microorganisms to use different substrates for performing a variety of biological processes (Wall et al., 2012), which was an important mechanism for soil microbial communities to respond to changes in the soil environment. Our results showed that the group capable of aromatic compound degradation was the main predictors of soil bacteria functional structure, then followed by the groups capable of aerobic chemoheterotrophy and chemoheterotrophy which were the predominant functional groups in soil (Figures 1C, 2B). These functional groups were related to the soil carbon cycle process (Liang et al., 2020), and had a significant correlation with the corresponding soil enzyme activity (Table 4), which indicated that changes in soil carbon pools may be the main factor driving soil bacterial functional structure (O’donnell et al., 2001). Soil bacterial functional groups were closely related to the diversity of above-ground plants. Rotation lead to changes in the functional groups of soil bacteria due to the high diversity of above-ground plants with a higher diversity of plant litters and root exudates input to soil (Schlatter et al., 2015), therefore, corn insertions treatments have a great impact on soil functional structure (Figure 1D). Nevertheless, NPK fertilization only had an effect on the amount of soil organic matter rather than diversity of organic matter thus showed little effect on soil functional structure (Figure 1D). The effect of corn insertions treatments on soil functions under fertilized and non-fertilized conditions was different, however, as the corn insertion treatments weakened soil nitrification and promoted other nitrogen cycle functions regardless of fertilization (Witt et al., 2000; Paungfoo-Lonhienne et al., 2017). The possible reason was that corn insertions caused an input which was varied from the original organic matter in the soil, moreover, the utilization of exogenous carbon was limited by nitrogen, therefore, microorganisms need to strengthen the nitrogen acquisition function to meet its own demand for nitrogen (Kuzyakov, 2010). The functional groups capable of carbon degradation were significantly positively correlated with microbial activity indicators (MBC, MBC:SOC, qCO_2_, BR, and SIR), which showed that the formation of different soil bacterial functional structures were closely related to the soil carbon sources and the response of microbial activity to different carbon sources (Preston-Mafham et al., 2002). The results of redundant analysis based on soil bacterial function and microbial activity indicators also confirmed this point (Figure 3B).

## Conclusion

Corn insertions by CS and CCS rotation and NPK fertilization treatments significantly changed the characteristics of farmland soil in Gongzhuling, which was also reflected in the changes of soil microbial activity, bacterial community structure, functional structure and diversity. we systematically demonstrated that bacterial community structure was significantly affected by fertilization and corn insertion of CCS, however, bacterial functional structure was only affected by corn insertion of CCS. Furthermore, because corn insertion of CCS with more corn continuous insertion into soybean field, the CCS weakened the legacy effect of soybean and then showed a significant difference in bacterial communities when compared to Sm. We found the SOC and AVK were main environmental factors affecting the bacterial community structure, the microbial quotient (MBC:SOC) and soil BR were the driving factors of soil bacterial functional structure. In addition, our research on microbial activity and bacterial functional groups indicated that corn insertions increased the activities of extracellular enzyme and then tend to promote the metabolic process of growth of living organisms. This promoted the process of soil carbon metabolism, the correlation between microbial activity parameters and bacterial functional groups had clearly corroborated this point. Above all, the variability of soil microbial, especially the functional structure of soil bacteria under corn insertions may be the main reason for corn insertions with higher soil productivity, which was similar to the effects of fertilization on soil productivity. This will lend insight into regulating of bacterial function structure by corn insertions with combination of appropriate fertilizer regime in a long-term soybean monocropping system.

## Data Availability Statement

The datasets generated for this study can be found in online repositories. The names of the repository/repositories and accession number(s) can be found below: https://www.ncbi.nlm.nih.gov/, PRJNA658343.

## Author Contributions

DR, FX, and WZ designed experiments. DR, FM, XYY, MZ, XDY, JZ, and QQ performed the experiments. DR, XDY, and KK analyzed the data. DR wrote the manuscript. FX, WZ, and KK revised the manuscript. All authors read and approved the final manuscript. All authors have read and agreed to the published version of the manuscript.

## Conflict of Interest

The authors declare that the research was conducted in the absence of any commercial or financial relationships that could be construed as a potential conflict of interest.

## Footnotes

1 http://www.loucalab.com/archive/FAPROTAX/lib/php/index.php?section=Home

2 https://www.ncbi.nlm.nih.gov/sra/

## Supplementary Material

Supplementary Figure 1 Spatial imagery of the studied location of the experimental area.

Supplementary Figure 2 Heatmap clustering analysis of the top 15 bacterial phyla **(A)** and the top 15 function groups **(B)**.

Supplementary Figure 3 Differences in relative abundance of bacterial phyla among all treatments. Error bars show 95% confidence intervals.

Supplementary Figure 4 Differences in relative abundance of bacterial functional groups among all treatments. Error bars show 95% confidence intervals.

Supplementary Table 1 Distribution of component crop in different corn insertions treatments from 2012 to 2017.

Supplementary Table 2 Permutational multivariate analysis of variance (PERMANOVA) results based on Bray Curtis distance dissimilarities of OTU level and relative abundance of functional groups.

Supplementary Table 3 Pearson’s correlation coefficients for soil properties, soil microbial parameters, extracellular enzyme activities and main bacteria phyla.

## Abbreviations

SmF: soybean continuous cropping with fertilization
CSF: soybean followed 1-year corn system with fertilization
CCSF: soybean followed 2-year corn system with fertilization
Smb: soybean continuous cropping with no fertilization
CSb: soybean followed 1-year corn system with no fertilization
CCSb: soybean followed 2-year corn system with no fertilization
IVE: invertase
BG: β -glucosidase
XYL: β -xylosidase
CBH: β -D-1,4-cellobiohydrolase
AVN: available nitrogen
AVP: available phosphorus
AVK: available potassium
MBC: microbial biomass carbon
NH_4_^+^-N: ammonium nitrogen
NO_3_^–^-N: nitrate nitrogen
SOC: soil organic carbon
DOC: dissolved organic carbon
TN: total nitrogen
WC: water content
qCO_2_: metabolic quotient
BR: basal respiration
SIR: soil induced respiration
CCE: soil carbon cycling enzyme activity.

## References

[B1] Amorim H. C. S. Ashworth A. J. Brye K. R. Wienhold B. J. Savin M. C. Owens P. R. (2020). Soil quality indices as affected by long-term burning, irrigation, tillage, and fertility management. *Soil Sci. Soc. Am. J.* 10.1002/saj2.20188 [Epub ahead of print].

[B2] Ahmad W. Shah Z. Jamal M. Shah K. A. (2014). Recovery of organic fertility in degraded soil through fertilization and crop rotation. *J. Saudi Soc. Agric. Sci.* 13 92–99. 10.1016/j.jssas.2013.01.007

[B3] Archer E. (2016). *rfPermute: Estimate Permutation p-Values for Random Forest Importance Metrics. R Package Version 1.*

[B4] Banerjee S. Walder F. Büchi L. Meyer M. Held A. Y. Gattinger A. (2019). Agricultural intensification reduces microbial network complexity and the abundance of keystone taxa in roots. *ISME J.* 13 1722–1736. 10.1038/s41396-019-0383-2 30850707 PMC6591126

[B5] Bardgett R. D. van der Putten W. H. (2014). Belowground biodiversity and ecosystem functioning. *Nature* 515 505–511. 10.1038/nature13855 25428498

[B6] Bell C. W. Fricks B. E. Rocca J. D. Steinweg J. M. McMahon S. K. Wallenstein M. D. (2013). High-throughput fluorometric measurement of potential soil extracellular enzyme activities. *J. Vis. Exp.* 2013:e50961. 10.3791/50961 24299913 PMC3991303

[B7] Bhatti A. A. Haq S. Bhat R. A. (2017). Actinomycetes benefaction role in soil and plant health. *Microb. Pathog.* 111 458–467. 10.1016/j.micpath.2017.09.036 28923606

[B8] Bray R. H. Kurtz L. T. (1945). Determination of total, organic, and available forms of phosphorus in soils. *Soil Sci.* 59 39–46. 10.1097/00010694-194501000-00006

[B9] Carney K. M. Matson P. A. (2005). Plant communities, soil microorganisms, and soil carbon cycling: does altering the world belowground matter to ecosystem functioning? *Ecosystems* 8 928–940. 10.1007/s10021-005-0047-0

[B10] Castle S. C. Sullivan B. W. Knelman J. Hood E. Nemergut D. R. Schmidt S. K. (2017). Nutrient limitation of soil microbial activity during the earliest stages of ecosystem development. *Oecologia* 185 513–524. 10.1007/s00442-017-3965-6 28983721

[B11] Chao A. Chazdon R. L. Colwell R. K. Shen T.-J. (2005). A new statistical approach for assessing similarity of species composition with incidence and abundance data. *Ecol. Lett.* 8 148–159. 10.1111/j.1461-0248.2004.00707.x

[B12] Chen J. Guo Q. Liu D. Hu C. Sun J. Wang X. (2020). Composition, predicted functions, and co-occurrence networks of fungal and bacterial communities_ Links to soil organic carbon under long-term fertilization in a rice-wheat cropping system. *Eur. J. Soil Biol.* 100:103226. 10.1016/j.ejsobi.2020.103226

[B13] Dai Z. Su W. Chen H. Barberán A. Zhao H. Yu M. (2018). Long-term nitrogen fertilization decreases bacterial diversity and favors the growth of Actinobacteria and *Proteobacteria* in agro-ecosystems across the globe. *Glob. Chang. Biol.* 24 3452–3461. 10.1111/gcb.14163 29645398

[B14] Derksen D. A. Lafond G. P. Thomas A. G. Loeppky H. A. Swanton C. J. (1993). Impact of agronomic practices on weed communities: tillage systems. *Weed Sci.* 41 409–417. 10.1017/S0043174500052127

[B15] Dilly O. Munch J.-C. (1998). Ratios between estimates of microbial biomass content and microbial activity in soils. *Biol. Fertil. Soils* 27 374–379. 10.1007/s003740050446

[B16] Fan F. Yin C. Tang Y. Li Z. Song A. Wakelin S. A. (2014). Probing potential microbial coupling of carbon and nitrogen cycling during decomposition of maize residue by 13C-DNA-SIP. *Soil Biol. Biochem.* 70 12–21. 10.1016/j.soilbio.2013.12.002

[B17] Ferreira M. C. Andrade D. de S. Chueire L. M. de O. Takemura S. M. (2000). Tillage method and crop rotation effects on the population sizes and diversity of bradyrhizobia nodulating soybean. *Soil Biol. Biochem.* 32 627–637. 10.1016/S0038-0717(99)00189-3

[B18] Fierer N. (2017). Embracing the unknown: disentangling the complexities of the soil microbiome. *Nat. Rev. Microbiol.* 15 579–590. 10.1038/nrmicro.2017.87 28824177

[B19] Gollner G. Starz W. Friedel J. K. (2019). Crop performance, biological N fixation and pre-crop effect of pea ideotypes in an organic farming system. *Nutr. Cycl. Agroecosyst.* 115 391–405. 10.1007/s10705-019-10021-4

[B20] Gopal M. Gupta A. Arunachalam V. Magu S. P. (2007). Impact of azadirachtin, an insecticidal allelochemical from neem on soil microflora, enzyme and respiratory activities. *Bioresour. Technol.* 98 3154–3158. 10.1016/j.biortech.2006.10.010 17166716

[B21] Grandy A. S. Robertson G. P. (2007). Land-use intensity effects on soil organic carbon accumulation rates and mechanisms. *Ecosystems* 10 59–74. 10.1007/s10021-006-9010-y

[B22] Hill T. C. Walsh K. A. Harris J. A. Moffett B. F. (2003). Using ecological diversity measures with bacterial communities. *FEMS Microbiol. Ecol.* 43 1–11. 10.1111/j.1574-6941.2003.tb01040.x 19719691

[B23] Kelly C. Schipanski M. Kondratieff B. Sherrod L. Schneekloth J. Fonte S. J. (2020). The effects of dryland cropping system intensity on soil function and associated changes in macrofauna communities. *Soil Sci. Soc. Am. J.* 84 1854–1870. 10.1002/saj2.20133

[B24] Kuzyakov Y. (2010). Priming effects: interactions between living and dead organic matter. *Soil Biol. Biochem.* 42 1363–1371. 10.1016/j.soilbio.2010.04.003

[B25] Li H. Zhang Y. Yang S. Wang Z. Feng X. Liu H. (2019). Variations in soil bacterial taxonomic profiles and putative functions in response to straw incorporation combined with N fertilization during the maize growing season. *Agric. Ecosyst. Environ.* 283:106578. 10.1016/j.agee.2019.106578

[B26] Liang S. Deng J. Jiang Y. Wu S. Zhou Y. Zhu W. (2020). Functional distribution of bacterial community under different land use patterns based on FaProTax function prediction. *Pol. J. Environ. Stud.* 29 1245–1261. 10.15244/pjoes/108510

[B27] Ling N. Chen D. Guo H. Wei J. Bai Y. Shen Q. (2017). Differential responses of soil bacterial communities to long-term N and P inputs in a semi-arid steppe. *Geoderma* 292 25–33. 10.1016/j.geoderma.2017.01.013

[B28] Liu Y.-R. Delgado-Baquerizo M. Trivedi P. He J.-Z. Wang J.-T. Singh B. K. (2017). Identity of biocrust species and microbial communities drive the response of soil multifunctionality to simulated global change. *Soil Biol. Biochem.* 107 208–217. 10.1016/j.soilbio.2016.12.003

[B29] Louca S. Parfrey L. W. Doebeli M. (2016). Decoupling function and taxonomy in the global ocean microbiome. *Science* 353 1272–1277. 10.1126/science.aaf4507 27634532

[B30] Lupwayi N. Z. Kanashiro D. A. Eastman A. H. Hao X. (2018). Soil phospholipid fatty acid biomarkers and β-glucosidase activities after long-term manure and fertilizer N applications. *Soil Sci. Soc. Am. J.* 82 343–353. 10.2136/sssaj2017.09.0340

[B31] Macholdt J. Piepho H.-P. Honermeier B. (2019). Does fertilization impact production risk and yield stability across an entire crop rotation? Insights from a long-term experiment. *Field Crops Res.* 238 82–92. 10.1016/j.fcr.2019.04.014

[B32] Mäder P. Fliessbach A. Dubois D. Gunst L. Fried P. Niggli U. (2002). Soil fertility and biodiversity in organic farming. *Science* 296 1694–1697. 10.1126/science.1071148 12040197

[B33] Maul J. E. Cavigelli M. A. Vinyard B. Buyer J. S. (2019). Cropping system history and crop rotation phase drive the abundance of soil denitrification genes nirK, nirS and nosZ in conventional and organic grain agroecosystems. *Agric. Ecosyst. Environ.* 273 95–106. 10.1016/j.agee.2018.11.022

[B34] McDaniel M. D. Grandy A. S. (2016). Soil microbial biomass and function are altered by 12 years of crop rotation. *Soil* 2 583–599. 10.5194/soil-2-583-2016

[B35] Moore-Kucera J. Dick R. P. (2008). PLFA profiling of microbial community structure and seasonal shifts in soils of a Douglas-fir chronosequence. *Microb. Ecol.* 55 500–511. 10.1007/s00248-007-9295-1 17786504

[B36] Munroe J. W. McCormick I. Deen W. Dunfield K. E. (2016). Effects of 30 years of crop rotation and tillage on bacterial and archaeal ammonia oxidizers. *J. Environ. Qual.* 45 940–948. 10.2134/jeq2015.06.0331 27136161

[B37] Navarro-Noya Y. E. Gómez-Acata S. Montoya-Ciriaco N. Rojas-Valdez A. Suárez-Arriaga M. C. Valenzuela-Encinas C. (2013). Relative impacts of tillage, residue management and crop-rotation on soil bacterial communities in a semi-arid agroecosystem. *Soil Biol. Biochem.* 65 86–95. 10.1016/j.soilbio.2013.05.009

[B38] O’donnell A. G. Seasman M. Macrae A. Waite I. Davies J. T. (2001). Plants and fertilisers as drivers of change in microbial community structure and function in soils. *Plant Soil* 232 135–145. 10.1023/A:1010394221729

[B39] Oksanen J. Blanchet F. G. Friendly M. Kindt R. Legendre P. McGlinn D. (2017). *Vegan: Community Ecology Package. 2017. R Package Version 2.4-5.*

[B40] Ouyang Y. Evans S. E. Friesen M. L. Tiemann L. K. (2018). Effect of nitrogen fertilization on the abundance of nitrogen cycling genes in agricultural soils: a meta-analysis of field studies. *Soil Biol. Biochem.* 127 71–78. 10.1016/j.soilbio.2018.08.024

[B41] Paungfoo-Lonhienne C. Wang W. Yeoh Y. K. Halpin N. (2017). Legume crop rotation suppressed nitrifying microbial community in a sugarcane cropping soil. *Sci. Rep.* 7 1–7. 10.1038/s41598-017-17080-z 29196695 PMC5711877

[B42] Preston-Mafham J. Boddy L. Randerson P. F. (2002). Analysis of microbial community functional diversity using sole-carbon-source utilisation profiles-a critique. *FEMS Microbiol. Ecol.* 42 1–14. 10.1111/j.1574-6941.2002.tb00990.x 19709261

[B43] Ramette A. Tiedje J. M. (2007). Multiscale responses of microbial life to spatial distance and environmental heterogeneity in a patchy ecosystem. *Proc. Natl. Acad. Sci. U.S.A.* 104 2761–2766. 10.1073/pnas.0610671104 17296935 PMC1815255

[B44] Ramirez K. S. Lauber C. L. Knight R. Bradford M. A. Fierer N. (2010). Consistent effects of nitrogen fertilization on soil bacterial communities in contrasting systems. *Ecology* 91 3463–3470. 10.1890/10-0426.1 21302816

[B45] Robertson G. P. Vitousek P. M. (2009). Nitrogen in agriculture: balancing the cost of an essential resource. *Annu. Rev. Environ. Resour.* 34 97–125. 10.1146/annurev.environ.032108.105046

[B46] Rousk J. Bååth E. Brookes P. C. Lauber C. L. Lozupone C. Caporaso J. G. (2010). Soil bacterial and fungal communities across a pH gradient in an arable soil. *ISME J.* 4 1340–1351. 10.1038/ismej.2010.58 20445636

[B47] Savci S. (2012). Investigation of effect of chemical fertilizers on environment. *Apcbee Proc.* 1 287–292. 10.1016/j.apcbee.2012.03.047

[B48] Schlatter D. C. Bakker M. G. Bradeen J. M. Kinkel L. L. (2015). Plant community richness and microbial interactions structure bacterial communities in soil. *Ecology* 96 134–142. 10.1890/13-1648.1 26236898

[B49] Sebilo M. Mayer B. Grably M. Billiou D. Mariotti A. (2004). The use of the “Ammonium Diffusion” method for δ15N-NH4+ and δ15N-NO3- measurements: comparison with other techniques. *Environ. Chem.* 1 99–103. 10.1071/EN04037

[B50] Segata N. Izard J. Waldron L. Gevers D. Miropolsky L. Garrett W. S. (2011). Metagenomic biomarker discovery and explanation. *Genome Biol.* 12:R60. 10.1186/gb-2011-12-6-r60 21702898 PMC3218848

[B51] Seifert C. A. Roberts M. J. Lobell D. B. (2017). Continuous corn and soybean yield penalties across hundreds of thousands of fields. *Agron. J.* 109 541–548. 10.2134/agronj2016.03.0134

[B52] Singh K. Trivedi P. Singh G. Singh B. Patra D. D. (2016). Effect of different leaf litters on carbon, nitrogen and microbial activities of sodic soils. *Land Degrad. Dev.* 27 1215–1226. 10.1002/ldr.2313

[B53] Storey J. D. Taylor J. E. Siegmund D. (2004). Strong control, conservative point estimation and simultaneous conservative consistency of false discovery rates: a unified approach. *J. R. Stat. Soc. Ser. B Stat. Methodol.* 66 187–205. 10.1111/j.1467-9868.2004.00439.x

[B54] Su J.-Q. Ding L.-J. Xue K. Yao H.-Y. Quensen J. Bai S.-J. (2015). Long-term balanced fertilization increases the soil microbial functional diversity in a phosphorus-limited paddy soil. *Mol. Ecol.* 24 136–150. 10.1111/mec.13010 25410123

[B55] Tiemann L. K. Grandy A. S. Atkinson E. E. Marin-Spiotta E. McDaniel M. D. (2015). Crop rotational diversity enhances belowground communities and functions in an agroecosystem. *Ecol. Lett.* 18 761–771. 10.1111/ele.12453 26011743

[B56] Vance E. D. Brookes P. C. Jenkinson D. S. (1987). An extraction method for measuring soil microbial biomass C. *Soil Biol. Biochem.* 19 703–707. 10.1016/0038-0717(87)90052-6

[B57] Walkley A. Black I. A. (1934). An examination of the Degtjareff method for determining soil organic matter, and a proposed modification of the chromic acid titration method. *Soil Sci.* 37 29–38. 10.1097/00010694-193401000-00003

[B58] Wall D. H. Behan-Pelletier V. Ritz K. Herrick J. E. Jones T. H. Six J. (2012). *Soil Ecology and Ecosystem Services.* Oxford: Oxford University Press. 10.1093/acprof:oso/9780199575923.001.0001

[B59] Walters W. Hyde E. R. Berg-Lyons D. Ackermann G. Humphrey G. Parada A. (2016). Improved bacterial 16S rRNA Gene (V4 and V4-5) and fungal internal transcribed spacer marker gene primers for microbial community surveys. *mSystems* 1:e009-15. 10.1128/mSystems.00009-15 27822518 PMC5069754

[B60] Witt C. Cassman K. G. Olk D. C. Biker U. Liboon S. P. Samson M. I. (2000). Crop rotation and residue management effects on carbon sequestration, nitrogen cycling and productivity of irrigated rice systems. *Plant Soil* 225 263–278. 10.1023/A:1026594118145

[B61] Zhang Y. Hao X. Alexander T. W. Thomas B. W. Shi X. Lupwayi N. Z. (2018). Long-term and legacy effects of manure application on soil microbial community composition. *Biol. Fertil. Soils* 54 269–283. 10.1007/s00374-017-1257-2

[B62] Zhang Y. Li T. Wu H. Bei S. Zhang J. Li X. (2019). Effect of different fertilization practices on soil microbial community in a wheat-maize rotation system. *Sustainability* 11:4088. 10.3390/su11154088

[B63] Zhao R. Chen X. Zhang F. (2009). Nitrogen cycling and balance in winter-wheat-summer-maize rotation system in Northern China Plain. *Acta Pedol. Sin.* 46 684–697.

[B64] Zhao X. Liu N. Guo X. Wang H. Sui B. Zhao L. (2017). Effect of straw and aluminum sulfate on soil organic-mineral complex and organic carbon distribution in light Chernozem. *J. Agro Environ. Sci.* 36 950–956.

[B65] Zhou J. Jiang X. Zhou B. Zhao B. Ma M. Guan D. (2016). Thirty four years of nitrogen fertilization decreases fungal diversity and alters fungal community composition in black soil in northeast China. *Soil Biol. Biochem.* 95 135–143. 10.1016/j.soilbio.2015.12.012

[B66] Zhu Y. Shi F. Zhang R. Wu Y. (2014). Comparison of bacterial diversity in rotational and continuous soybean cropping soils in Heilongjiang. *Acta Phytophylac. Sin.* 41 403–409.

