# Supplementary material for: Changes in Soil Microbial Activity, Bacterial Community Composition and Function in a Long-Term Continuous Soybean Cropping System After Corn Insertion and Fertilization
Source: Front Microbiol. 2021 Apr 7;12:638326. doi: 10.3389/fmicb.2021.638326 (PMC8059791; doi:10.3389/fmicb.2021.638326)
Supplement: Supplementary Figure 1 — Spatial imagery of the studied location of the experimental area. [file Data_Sheet_2.docx]

Supplementary Material


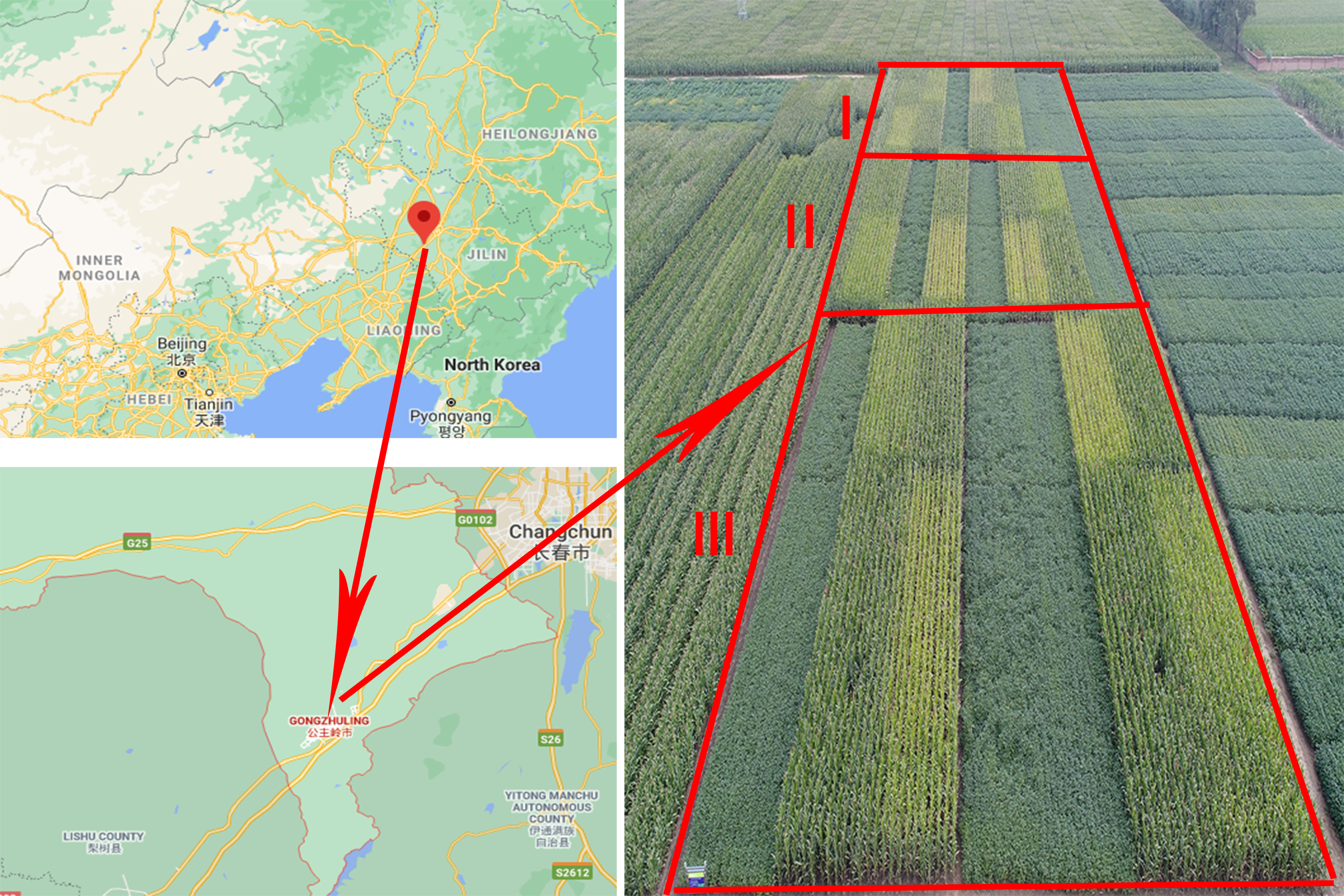


**Figure S1.** Spatial imagery of the studied location of the experimental area.


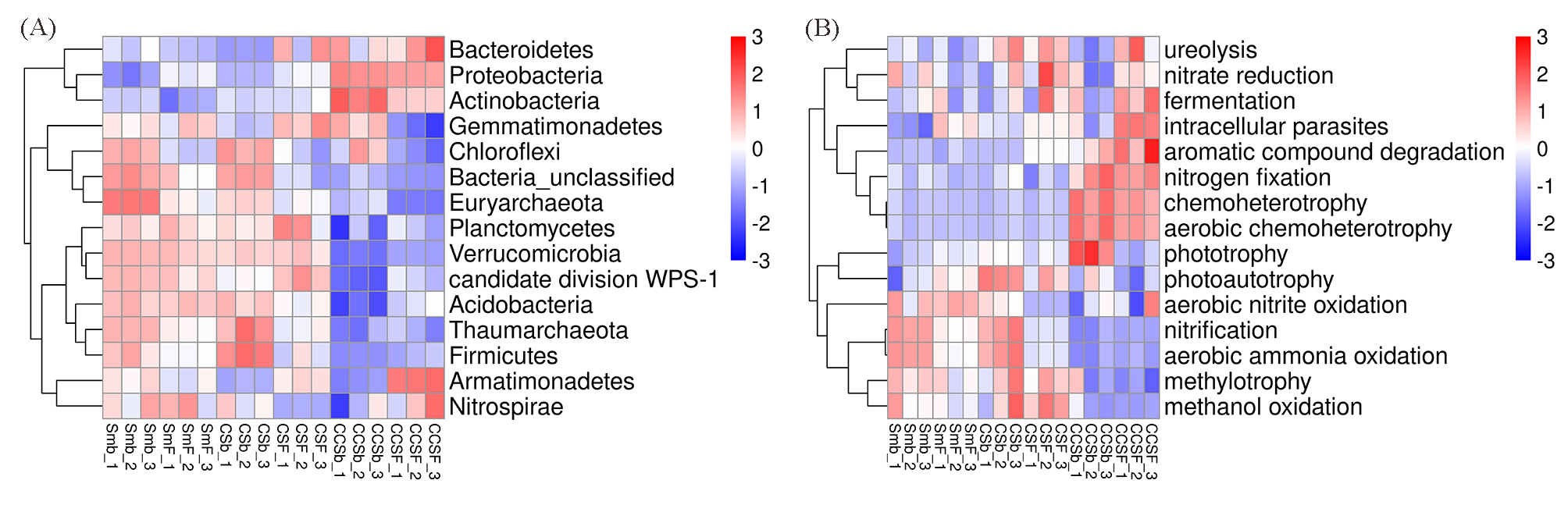


**Fig. S2.** Heatmap clustering analysis of the top 15 bacterial phyla (A) and the top 15 functuon groups (B).





**Fig. S3.** Differences in relative abundance of bacterial phyla among all treatments. Error bars show 95% confidence intervals.





**Fig. S4.** Differences in relative abundance of bacterial functional groups among all treatments. Error bars show 95% confidence intervals.
