# Supplementary material for: Changes in Soil Microbial Activity, Bacterial Community Composition and Function in a Long-Term Continuous Soybean Cropping System After Corn Insertion and Fertilization
Source: Front Microbiol. 2021 Apr 7;12:638326. doi: 10.3389/fmicb.2021.638326 (PMC8059791; doi:10.3389/fmicb.2021.638326)

Supplementary Material

**Table S1. Distribution of component crop in different corn insertions treatments from 2012 to 2017.**

| **2002-2011** | **Treat（2012-2017）** | **2012** | **2013** | **2014** | **2015** | **2016** | **2017(Sampling)** |
| --- | --- | --- | --- | --- | --- | --- | --- |
| **Ten years continuous soybean with conventional field management measures** | **Sm with NPK** | soybean | soybean | soybean | soybean | soybean | soybean |
|  | **CS with NPK** | corn | soybean | corn | soybean | corn | soybean |
|  | **CCS with** **NPK** | corn | corn | soybean | corn | corn | soybean |
|  | **Sm with no NPK** | soybean | soybean | soybean | soybean | soybean | soybean |
|  | **CS with no NPK** | corn | soybean | corn | soybean | corn | soybean |
|  | **CCS with no NPK** | corn | corn | soybean | corn | corn | soybean |

**Table S2. Permutational multivariate analysis of variance (PERMANOVA) results based on [Bray Curtis distance](C:/Users/Administrator/AppData/Local/youdao/dict/Application/8.9.3.0/resultui/html/index.html#/javascript:;) dissimilarities of OTU level and relative abundance of functional groups.**

|  | **Treatment** | **Df** | **Sums of squares** | **Mean squares** | **F.Model** | **Variation (R^2^)** | **Pr (>F)** |
| --- | --- | --- | --- | --- | --- | --- | --- |
| **Based on OTU** | **Fertilization** | 1 | 0.007152792 | 0.007152792 | 15.63059134 | 0.042034564 | **0.002**** |
|  | **Corn insertion** | 2 | 0.134693404 | 0.067346702 | 147.1689282 | 0.791548019 | **0.001**** |
|  | **Interaction** | 2 | 0.022826964 | 0.011413482 | 24.94123445 | 0.134146419 | **0.001**** |
| **Based on functions** | **Fertilization** | 1 | 0.001606967 | 0.001606967 | 1.662664105 | 0.035204429 | 0.231 |
|  | **Corn insertion** | 2 | 0.029067576 | 0.014533788 | 15.03752224 | 0.636794142 | **0.003**** |
|  | **Interaction** | 2 | 0.003374177 | 0.001687089 | 1.745562442 | 0.073919355 | 0.184 |

**Note** * P < 0.05; ** P < 0.01.

**Table S3. Pearson’s correlation coefficients for soil properties , soil microbial parameters, extracellular enzyme activities and main bacteria** **plyla.**

|  | **AVP** | **AVK** | **NH_4_^+^-N** | **NO_3_^−^-N** | **DOC** | **SOC** | **TN** | **C:N** | **PH** | **WC %** |
| --- | --- | --- | --- | --- | --- | --- | --- | --- | --- | --- |
| ***Proteobacteri*a** | 0.081 | -0.192 | 0.112 | 0.468 | 0.583* | 0.464 | 0.31 | 0.125 | -0.184 | 0.286 |
| ***Acidobacteria*** | 0.212 | 0.382 | 0.037 | -0.3 | -0.366 | -0.23 | -0.047 | -0.22 | -0.084 | -0.123 |
| ***Actinobacteria*** | -0.431 | -0.590** | 0.004 | 0.128 | 0.254 | 0.091 | -0.127 | 0.299 | 0.267 | 0.026 |
| ***Verrucomicrobia*** | 0.146 | 0.395 | -0.135 | -0.317 | -0.475* | -0.346 | -0.127 | -0.25 | 0.004 | -0.184 |
| ***Thaumarchaeota*** | -0.331 | -0.036 | -0.02 | -0.527* | -0.588* | -0.497* | -0.509* | 0.186 | 0.407 | -0.194 |
| ***Chloroflexi*** | -0.789** | -0.359 | -0.165 | -0.723** | -0.737** | -0.761** | -0.822** | 0.276 | 0.752** | -0.286 |
| **chao1** | -0.755** | -0.653** | -0.031 | -0.705** | -0.656** | -0.742** | -0.659** | 0.019 | 0.714** | -0.307 |
| **shannon** | -0.698** | -0.46 | -0.286 | -0.589* | -0.590** | -0.699** | -0.605** | -0.005 | 0.733** | -0.309 |
| **MBC** | 0.536* | 0.153 | 0.186 | 0.904** | 0.950** | 0.962** | 0.699** | 0.193 | -0.691** | 0.375 |
| **MBC:SOC** | 0.282 | -0.08 | 0.187 | 0.786** | 0.899** | 0.805** | 0.496* | 0.302 | -0.472* | 0.38 |
| **qCO_2_** | 0.346 | 0.363 | -0.073 | -0.284 | -0.408 | -0.334 | 0.138 | -0.672** | -0.119 | -0.119 |
| **BR** | 0.716** | 0.311 | 0.2 | 0.924** | 0.927** | 0.973** | 0.830** | 0.001 | -0.825** | 0.399 |
| **SIR** | 0.811** | 0.442 | 0.23 | 0.879** | 0.889** | 0.936** | 0.873** | -0.12 | -0.869** | 0.42 |
| **QR** | 0.798** | 0.540* | 0.284 | 0.710** | 0.739** | 0.770** | 0.797** | -0.22 | -0.804** | 0.446 |
| **IVE** | 0.695** | 0.44 | 0.534* | 0.746** | 0.803** | 0.835** | 0.767** | -0.053 | -0.766** | 0.620** |
| **BG** | 0.627** | 0.265 | 0.339 | 0.799** | 0.880** | 0.879** | 0.769** | -0.029 | -0.689** | 0.502* |
| **XYL** | 0.730** | 0.43 | 0.363 | 0.755** | 0.805** | 0.854** | 0.785** | -0.08 | -0.799** | 0.483* |
| **CBH** | 0.515* | 0.168 | 0.417 | 0.743** | 0.822** | 0.857** | 0.677** | 0.11 | -0.602** | 0.544* |

**Notes** MBC:[microbial](C:/Users/Administrator/AppData/Local/youdao/dict/Application/8.9.3.0/resultui/html/index.html#/javascript:;) [biomass](C:/Users/Administrator/AppData/Local/youdao/dict/Application/8.9.3.0/resultui/html/index.html#/javascript:;) [carbon](C:/Users/Administrator/AppData/Local/youdao/dict/Application/8.9.3.0/resultui/html/index.html#/javascript:;); CCE: the PC1 of principal coordinate analysis of IVE, BG, XYL and CHB which was defined as carbon metabolism enzyme activity; * P < 0.05; ** P < 0.01.


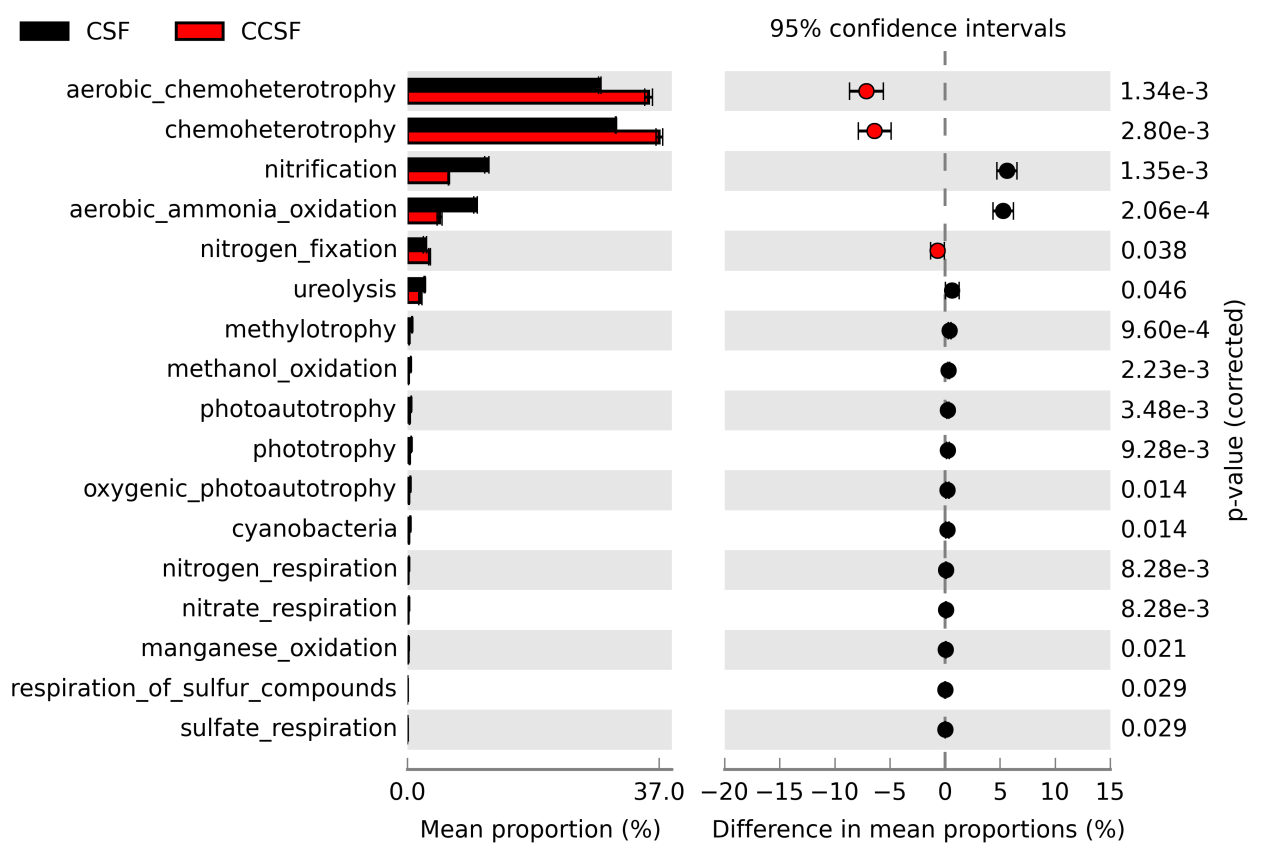

Supplement: Supplementary Table 1 — Distribution of component crop in different corn insertions treatments from 2012 to 2017. [file Data_Sheet_1.docx]
